# Harmine Alleviates Titanium Particle-Induced Inflammatory Bone Destruction by Immunomodulatory Effect on the Macrophage Polarization and Subsequent Osteogenic Differentiation

**DOI:** 10.3389/fimmu.2021.657687

**Published:** 2021-05-17

**Authors:** Liangliang Wang, Qing Wang, Wei Wang, Gaoran Ge, Nanwei Xu, Dong Zheng, Shijie Jiang, Gongyin Zhao, Yaozeng Xu, Yuji Wang, Ruixia Zhu, Dechun Geng

**Affiliations:** ^1^ Department of Orthopaedics, The Affiliated Changzhou No. 2 People’s Hospital of Nanjing Medical University, Changzhou, China; ^2^ Department of Orthopaedics, The First Affiliated Hospital of Soochow University, Suzhou, China; ^3^ Departments of Orthopedic Surgery and Biochemistry and Molecular Biology, Mayo Clinic, Rochester, MN, United States; ^4^ Department of Orthopedics, The Third Affiliated Hospital of Gansu University of Chinese Medicine, Baiyin, China

**Keywords:** peri-prosthetic osteolysis, chronic inflammation, harmine, macrophage polarization, osteoimmunomodulatory

## Abstract

Peri-prosthetic osteolysis (PPO) and following aseptic loosening are regarded as the prime reasons for implant failure after joint replacement. Increasing evidence indicated that wear-debris-irritated inflammatory response and macrophage polarization state play essential roles in this osteolytic process. Harmine, a β-carboline alkaloid primitively extracted from the *Peganum harmala* seeds, has been reported to have various pharmacological effects on monoamine oxidase action, insulin intake, vasodilatation and central nervous systems. However, the impact of harmine on debris-induced osteolysis has not been demonstrated, and whether harmine participates in regulating macrophage polarization and subsequent osteogenic differentiation in particle-irritated osteolysis remains unknown. In the present study, we investigated the effect of harmine on titanium (Ti) particle-induced osteolysis *in vivo* and *in vitro*. The results suggested harmine notably alleviated Ti particle-induced bone resorption in a murine PPO model. Harmine was also found to suppress the particle-induced inflammatory response and shift the polarization of macrophages from M1 phenotypes to M2 phenotypes *in vivo* and *in vitro*, which improved anti-inflammatory and bone-related cytokines levels. In the conditioned medium from Ti particle-stimulated murine macrophage RAW264.7 cells treated with harmine, the osteoblast differentiation ability of mouse pre-osteoblastic MC3T3-E1 cells was greatly increased. And we also provided evidences that the immunomodulatory capacity of harmine might be attributed to the inhibition of the c-Jun N-terminal kinase (JNK) in wear particle-treated macrophages. All the results strongly show that harmine might be a promising therapeutic agent to treat PPO.

## Introduction

Total joint arthroplasty (TJA) is becoming more successful and minimally invasive to treat severe joint disease (1, 2). TJA relieves pain symptoms effectively and improves the quality of patients’ daily life. However, despite advancement in surgical skills, materials and implant designs, a risk of aseptic loosening is still exist, which leads to peri-prosthetic osteolysis (PPO). Increasing evidence shows that peri-prosthetic inflammatory bone destruction initiated by debris mainly leads to arthroplasty failure (3, 4).

Wear debris shed from joint prostheses is generated by mechanical impact and biological responses, besides polymeric, metallic, ceramic debris, etc. (5–7). These particles induce a chronic inflammation response by the further recruitment of macrophages, which secrete multiple proinflammatory factors, such as chemokines, and cytokines (8, 9). Macrophages are critical in the periprosthetic inflammatory process. Moreover, increasing evidence has demonstrated that macrophage polarization exerts great influence in the pathogenesis of inflammatory osteolysis (10–12). For the past few years, the concept of M1 macrophages (marked by CD86 and inducible nitric oxide synthase (iNOS)) and M2 macrophages (marked by CD206 and arginase-1 (Arg-1)) has been built (13). M1 macrophages can be irritated by microbial products or proinflammatory cytokines. On the other hand, M1 macrophages will enhance phagocytic activity and improve proinflammatory cytokines generation to accelerate inflammation. On the contrary, M2 macrophages can produce multiple anti-inflammatory cytokines to reduce inflammatory response and promote wound healing (14). It has been proved that wear particles around the prosthesis enhance M1 macrophage expression and augment local inflammation (15). Low expression of M2 phenotype was found in the process. As previously reported M2 macrophages-induced local microenvironment promotes osseointegration and angiogenesis, which is known as osteoimmunology (16–18). Regulation of the macrophage polarization may bring benefits for attenuating wear debris-irritated inflammation (19, 20). As a consequence, it is meaningful to modulate macrophages from M1 phenotypes to M2 phenotypes to reduce debris-irritated osteolysis and strengthen prostheses osseointegration.

Harmine, a β-carboline alkaloid, is primitively extracted from the *Peganum harmala* seeds and then was found in diverse plants, animals and humans (21). It has been reported to have various pharmacological effects on monoamine oxidase action, insulin intake, vasodilatation and cerebral disease (22–25). Harmine has been reported to reduce bone loss of osteoporotic mice and affect bone metabolism. Furthermore, it has been reported that harmine inhibits osteoclast differentiation and bone resorption, promotes osteoblast differentiation through bone morphogenetic protein signaling pathway (26–28). Nevertheless, to this day, the effect of harmine on PPO has not been demonstrated. In addition, the effect of harmine on regulating inflammation and macrophage has not been explored. Besides, whether harmine participates in regulating macrophage polarization and subsequent osteogenic differentiation in debris-irritated osteolysis remains unknown.

Therefore, the experiment aimed to explore latent immunomodulatory ability of harmine on wear debris-treated macrophages and its succedent effect on osteogenesis. Interestingly, our evidence suggested that harmine administration can alleviate wear-debris-irritated inflammatory response and promote osteogenic differentiation in the conditioned medium. The influence of harmine on macrophage polarization might be the major cause of the immunomodulatory functions. This study suggested that harmine may be a hopeful therapeutic agent to treat PPO.

## Materials and Methods

### Particle Preparation

Ti particles were applied to produce osteolysis and the mean particle size was 1.64 ± 1.43 μm (95% <4 μm). For removal of endotoxins, prepared particles were heated at 190°C for 8 h and soaked in 70% ethanol for 2 days. After washing with sterile PBS, the particles were dried for the experiment.

### Animals

Experimental procedures stringently followed world admitted principles and gained approval from the ethics committee of our institute. Briefly, forty 7-week-old male C57BL/J6 mice were separated at random into four groups (10 per group): the sham operation group (sham), PBS with Ti particles (vehicle), and Ti particles together with low- (5 mg/kg/day) or high- (10 mg/kg/day) concentration of harmine. In this study, PPO model was established by placing Ti particles onto mice calvaria. After anesthetization, a sagittal incision was cut at the medium site of mouse calvaria. The isometric PBS was injected under the periostium in the sham group and the incision was sutured with no intervention. While in the other groups, 30 mg Ti particles were embedded under the periosteum at middle site of the calvaria. Harmine emulsion was prepared as previously reported (26). On the second day after operation, harmine emulsion at different concentration was intragastrically offered to mice in the low- and high-harmine groups. While in the other groups, mice were treated with isometric volume of emulsion without harmine. After daily administration of harmine for 2 weeks, the calvariae were collected and fixed for subsequent radiological and histological study. Besides, the major organs (liver and kidney) were obtained to evaluate the potential organ damage by harmine. At the same time, the blood samples were collected for biochemical index evaluation.

### Microcomputed Tomography (μCT) Analysis

Fixed calvaria were analyzed by μCT (SkyScan, Aartselaar, Belgium). The scanning parameters were set as a resolution of 9 μm with X-ray energy of 50 kV and 500 μA. Three-dimensional (3D) and two-dimensional (2D) images were reconstructed using the manufacturer’s software. Then a columniform region of interest (ROI, 3 × 3 × 1 mm) was drawn at the midline suture center to determine bone mineral density (BMD), number of pores, and bone volume/tissue volume (BV/TV) by analyzer software as described (29).

### Histological Analysis, Immunohistochemistry and Immunofluorescence Staining

The fixed calvaria were decalcified and paraffin embedded with the standard procedure. Coronal sections (5 μm) were made with a microtome and saved for hematoxylin and eosin (H&E) staining. Section pictures of staining were observed using a high-quality microscope. As previously described, bone thickness was analyzed according to the protocols (30). Masson trichrome (MT) staining kit was used to identify new immature collagen (blue), which indicated new bone and fibrous tissue formation. On the other hand, zones full of mature collagen emerged vivid red. In each section, positive regions were determined microscopically and histomorphometric analysis was performed using a Zeiss microscope by two independent observers.

Immunohistochemistry staining was conducted to identify interleukin (IL)-1β (1:500), tumor necrosis factor-α (TNF-α, 1:500), IL-6 (1:600), osteocalcin (OCN, 1:100) and Runt-related transcription factor 2 (Runx2, 1:500) (all purchased from Abcam, Cambridge, UK) expression. After antigen retrieval, sections were incubated with respective primary antibodies overnight at 4°C. Then sections were washed followed by 30 min incubation of the secondary antibody at normal temperature. Color was developed using 3, 3′-diaminobezidine tetrahydrochloride and rinsed sections were counterstained by hematoxylin. The positive cells which appeared brown or yellow in specific regions were counted using a microscope by two independent observers.

Immunofluorescence staining of tissue sections was conducted to evaluate the effect of harmine on macrophage polarization. After antigen retrieval, tissue sections were incubated with antibodies against anti-F4/80 (macrophage, 1:50) and either anti-iNOS (M1 phenotype, 1:100) or anti-Arg-1 (M2 phenotype, 1:1,000) at 4°C overnight. Respective goat anti-rat IgG H&L (Alexa Fluor 488; Abcam) or goat anti-rabbit IgG H&L (Alexa Fluor 647, Abcam) were applied for 30 min incubation at 37°C. DAPI was used to counterstain the nucleus. Finally, confocal microscopic pictures were obtained using a Zeiss laser scanning microscope (LSM 510; Zeiss). Image analysis was performed with the LSM 5 Release 4.2 software.

### Cell Culture

Murine macrophage RAW 264.7 cells were cultured in Dulbecco’s modified Eagle’s medium (DMEM) and pre-osteoblastic MC3T3-E1 cells were cultured in α-modified Eagle’s medium (α-MEM), both supplemented with 10% fetal bovine serum (FBS), 1% penicillin and streptomycin in a 90% humidity incubator of 5% CO2 at 37°C. Cells were added to various plates before treatment without or with Ti particles (0.1 mg/ml) or a mixture of various concentrations of harmine (10 and 50 μM) and Ti particles.

### Cell Viability Assay

The proliferation of RAW 264.7 cells was investigated by a cell counting kit-8 (CCK-8) assay. RAW 264.7 cells (1.0 × 10^4^ cells/well) were cultured in triplicate for 48 h. Medium with 10% CCK-8 buffer was applied. The optical density of experimental samples was detected at 450 nm using a microplate-reader (BioTek, Winooski VT, USA) and cell viability was studied. In addition, the cell growth inhibition rate which reflects the extent to which the cell proliferation ability is inhibited compared to the control was converted based on the CCK-8 data (31). In detail, the calculation formula is [growth inhibition rate = (1 − A450 nm value of the drug group/A450 nm value of the control group) × 100%].

### Immunofluorescence Assay of Macrophage Polarization *In Vitro*

In order to study iNOS and Arg-1 expression by harmine, RAW 264.7 cells were added to 24-well plates (1 × 10^5^ cells/well). Bone marrow derived macrophage cells (BMDM) were also used and isolated from the mice as previously described (32). The cells were both treated with Ti particles with or without various concentrations of harmine (10 and 50 μM) for 24 h. Then cells were fixed by 4% paraformaldehyde at normal temperature. 10% FBS was used to block the nonspecific binding sites. Immunofluorescence assay was then performed according to the identical protocol depicted above.

### Nitric Oxide (NO) Production Assay

As described above, Ti particles (0.1 mg/ml) or a mixture of various concentrations of harmine (10 and 50 μM) and Ti particles were added to 24-well plates (1 × 10^5^ cells/well) and cultured for 4 h. NO production in the culture supernatant from each well was measured with a commercial NO assay kit (S0021, Beyotime, Shanghai, China) according to the manufacturer’s protocol, which is based on the Griess reaction. In short, 50 μl cell culture medium was mixed with 100 μl Griess reagents I and II at normal temperature in a 96-well plate, and reacted for 10 min. The absorbance was determined by a microplate reader at 540 nm.

### Enzyme-Linked Immunosorbent Assay (ELISA)

To better understand the effect of harmine on inflammation, ELISA was conducted to assess the level of inflammatory factors. RAW 264.7 cells were cultured for 96 h and cell medium was centrifuged at 3,000 rpm for 10 min. The supernatants were detected by a standard ELISA kit (R&D systems, Minnesota, USA). The optical densities were determined by a micro ELISA plate reader at 450 nm.

### Real-Time Polymerase Chain Reaction (RT-PCR)

Total RNA was collected from cells using the NucleoSpin RNA Kit (MN, Düren, Germany) following the manufacturer’s method and reverse-transcribed by the High-Capacity cDNA Reverse Transcription Kit (Applied Biosystems, Foster City, CA, USA). All reactions were run three times independently. In addition, β-actin was used as the housekeeping gene. The primer sequences in this section were as follows:

CD86 forward 5’-TGGGCGCAGAGAAACTTGAT-3’ and reverse 5’-AAGCCCGTGTCCTTGATCTG-3’;
CD163 forward 5’-GTGGTCAACTCCGCTTGGTA-3’ and reverse 5’-CTTGGGGCACCATCTGTGAT-3’;
BMP-2 forward 5’-AACGAGAAAAGCGTCAAGCC-3’ and reverse 5’-AGGTGCCACGATCCAGTCAT-3’;
VEGF forward 5’-GCAAGAGAAGACACGGTGGT-3’ and reverse 5’-CAGGAGGTGGGGTAAGGAG-3’;
OPN forward 5’-CACTCCAATCGTCCCTACAGT-3’ and reverse 5’-CTGGAAACTCCTAGACTTTGACC-3’;
Runx2 forward 5’-TTGACCTTTGTCCCAATGC-3’ and reverse 5’-AGGTTGGAGGCACACATAGG-3’;
OCN forward 5’-TTGAACTGTTTGTTTTGGACCC-3’ and reverse 5’-CCAACAGACACCAGTTGTAAAG-3’;
Osterix forward 5’-TGAGCTGGAACGTCACGTGC-3’ and reverse 5’-AAGAGGAGGCCAGCCAGACA-3’;
β-actin forward 5’-GTGACGTTGACATCCGTAAAGA-3’ and reverse 5’-GTAACAGTCCGCCTAGAAGCAC-3’.

### Osteogenic Differentiation Effect of Pre-Osteoblastic MC3T3-E1 Cells in Conditioned Medium (CM)

The conditioned medium was obtained from RAW 264.7 cells in different groups described above. After 4 days of culture in DMEM, the CM from different groups were collected and centrifuged to obtain supernatants. Then the supernatants were mixed with DMEM at a 1:2 ratio. Pre-osteoblastic MC3T3-E1 cells were seeded onto a 24-well plate (5.0 × 10^4^ cells) and cultured in DMEM. And after incubation for 12 h, the medium was replaced by CM for further incubation. Correspondingly, the CM was changed every 48 h.

### Alkaline Phosphatase (ALP) Staining

ALP staining of MC3T3-E1 cells in conditioned medium was performed after 7 days of culture. In brief, after 8 min fixation, cells were rinsed and incubated in BCIP/NBT working solution (Beyotime) for 10 min at 37°C. The pictures of staining in culture dishes were obtained.

### Alizarin Red S (ARS) Staining

The mineralization of MC3T3-E1 cells was detected by ARS staining. Briefly, MC3T3-E1 cells were cultured in conditioned medium for 21 days and then rinsed with PBS followed by 8 min fixation. Cells were then incubated for 5 min with ARS solution (Cyagen Biosciences). To quantify the mineralization, the stained layers were solubilized with 10% cetylpyridinium chloride (Sigma). Dye absorbance was detected at 490 nm.

### Immunofluorescence Staining of Runx2

Pre-osteoblastic MC3T3-E1 cells were cultured in CM and fixed in 4% paraformaldehyde. After 15 min permeabilization by 0.1% Triton-X, cells were blocked by 1% BSA and incubated at 4°C with Runx2 (1:500, Abcam) antibodies overnight. After washing with PBS, cells were incubated with DY-554 phalloidin. Immunofluorescence assay was then performed according to the identical protocol described above. The confocal microscopic pictures were obtained using a Zeiss laser scanning microscope.

### Western Blot Analysis

The RAW264.7 cells (5 × 10^5^ cells/well) were pretreated with 50 μM harmine for 4 h followed with the addition of Ti particles. Cells were lysed and boiled by sodium dodecyl sulfate (SDS) Lamini buffer. SDS-polyacrylamide gel electrophoresis was performed on 10% polyacrylamide gel and transferred to polyvinylidene fluoride membrane. These antibodies were detected in western blotting: JNK, P-JNK, p65, P-p65, ERK, P-ERK, p38, P-p38, iNOS, CD206 and β-actin (all 1:1,000 dilution, Abcam, UK). The stained protein bands were obtained by an ECL detection kit (Sigma-Aldrich).

### Statistical Analysis

SPSS 17.0 software was applied for data analysis. Results were shown as mean ± SD. The significance of the differences was measured by one-way analysis of variance (ANOVA) with Tukey *post hoc* multiple comparison tests. A *p* value < 0.05 represented statistical significance.

## Results

### Harmine Alleviated Ti Particle-Induced Osteolysis and Bone Loss *In Vivo*

**Figure 1:**
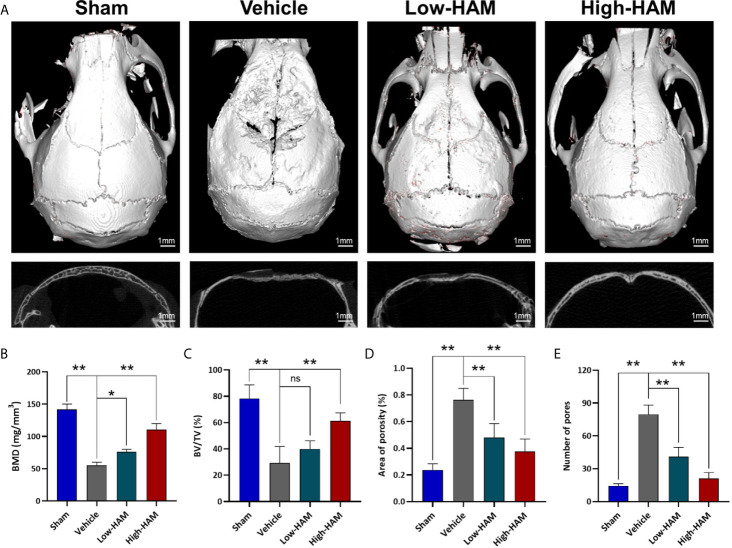
Harmine alleviated Ti debris-stimulated osteolysis and bone loss. **(A)** Representative micro-CT 3D and 2D pictures of murine calvaria. Scale bar indicates 1 mm. Computations for **(B)** BMD, **(C)** BV/TV, **(D)** Area of porosity and **(E)** Number of pores. n = 3. All data were expressed as the mean ± SD, ns, no significance, *p < 0.05, **p < 0.01, compared with the vehicle group.

In order to study the effect of harmine on osteolysis, micro-CT was performed to obtain 3D reconstruction and 2D fault pictures. Micro-CT results showed severe bone resorption distributed mostly at the middle site of calvaria in the Ti group, while there was less in the sham group (**Figure 1A**). In the harmine-treatment groups, debris-irritated osteolysis was alleviated in a dose-dependent manner, whereby less bone resorption was found by treatment of higher concentration of harmine, compared with the low harmine-treatment group. Quantitative analysis showed declines of 60.5% in BMD and 62.5% in BV/TV, and a 3.2-fold increase in the number of pores in the presence of Ti particles compared with the sham group (p <0.01). When harmine was intragastrically administrated at 5 or 10 mg/kg/day, BMD and BV/TV were greatly upregulated compared with the vehicle group (**Figures 1B, C**). Correspondingly, harmine also reduced area of porosity and number of pores (**Figures 1D, E**).

### Harmine Reduced the Inflammatory Infiltrate and Cytokines Expression *In Vivo*

**Figure 2:**
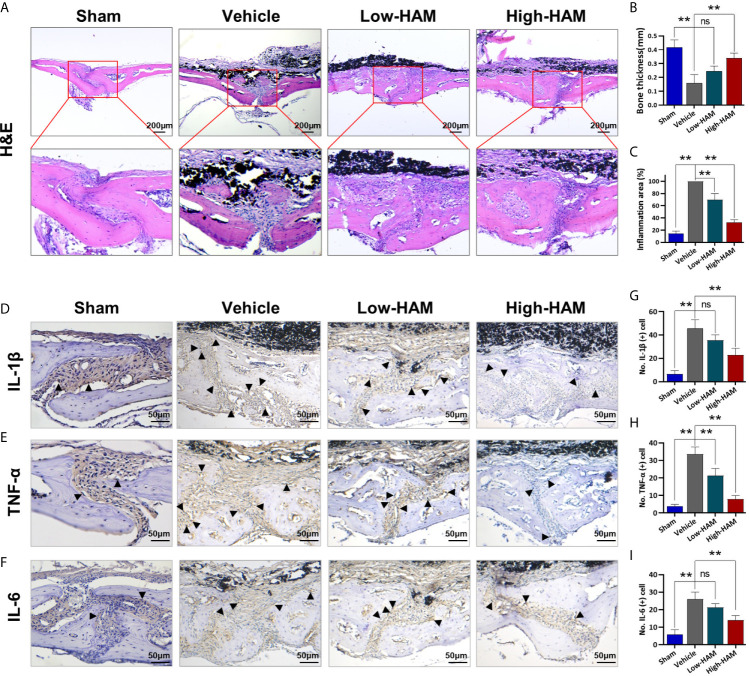
Harmine reduced the inflammatory infiltrate and cytokines expression. **(A)** Histological staining of (H&E). **(B)** Bone thickness. **(C)** Inflammation area. Immunohistochemical staining of inflammatory factors: **(D)** IL-1β, **(E)** TNF-α, and **(F)** IL-6. Number of positive cells: **(G)** IL-1β, **(H)** TNF-α, and **(I)** IL-6. Scale bar indicates 200 and 50 μm. n = 3. All data were expressed as the mean ± SD, ns. no significance, **p < 0.01, compared with the vehicle group.

To further study the mechanisms underlying the effect of harmine on PPO, wear particle-induced local inflammation and inflammatory cytokines production was measured. Consistent with micro-CT, H&E staining further demonstrated that harmine could inhibit debris-induced bone resorption *in vivo* (**Figure 2A**). Pictures of H&E staining showed few bone pittings in the sham group, whereas clear osteolysis appeared in the vehicle group (**Figure 2A**). In the Ti group, histomorphometric analysis revealed a 62.5% decrease in bone thickness and a 3.9-fold increase in inflammation area, compared with the sham group (**Figures 2B, C**). Harmine distinctly impacted these Ti-particle-induced effects. In addition, harmine toxicity was not found *in vivo* based on the H&E staining of the major organs and biochemical index evaluation of blood samples (**Figure S1**). As expected, addition of harmine increased the bone thickness and reduced inflammation area. Immunohistochemical staining of calvarial tissue showed positive stainings for IL-1β, TNF-α, IL-6 were distributed mainly in the inflammatory cells around the eroded areas (**Figures 2D–F**). And levels of IL-1β, TNF-α, and IL-6 were greatly elevated in the presence of Ti particles, compared with the sham group (**Figures 2D–I**). On the contrary, few positive staining responses for these inflammatory cytokines were observed in the low and high harmine-treatment groups.

### Harmine Inhibited Particle-Induced Inflammatory Reactions by Regulating Macrophage Polarization *In Vivo*

**Figure 3:**
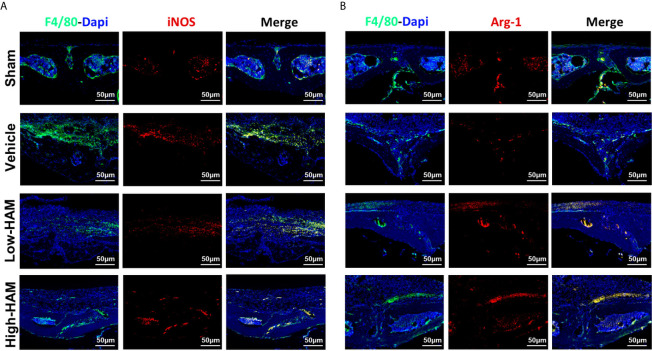
Harmine treatment shifted the polarization of macrophages from M1 to M2 in PPO. Representative immunofluorescent staining images for **(A)** iNOS (M1 marker) and **(B)** Arg-1 (M2 marker). Scale bar indicates 50 μm. n = 3.

Macrophages have the ability to polarize into diverse phenotypes in the multiple microenvironments. Specifically, M1 phenotype is proinflammatory, while M2 phenotype is anti-inflammatory. Considering the vital role of macrophages in the inflammatory response, the relationship between harmine and macrophage polarization was investigated *in vivo*. It turned out that massive M1 phenotype macrophages (F4/80^+^/iNOS^+^, merged as orange) distributed around the eroded areas in the vehicle group, and just a few expressed the M2 phenotype (F4/80^+^/Arg-1^+^, merged as orange) (**Figures 3A, B**). Nevertheless, penetration of F4/80^+^/iNOS^+^-positive M1 phenotype macrophages is reversed by harmine and the proportion of anti-inflammatory F4/80^+^/Arg-1^+^-positive M2 phenotype macrophages increases. This indicated harmine treatment shifted the polarization of macrophages from M1 to M2, thereby decreased inflammation in PPO.

### Harmine Promotes Osteogenic Formation in the Murine PPO Model

**Figure 4:**
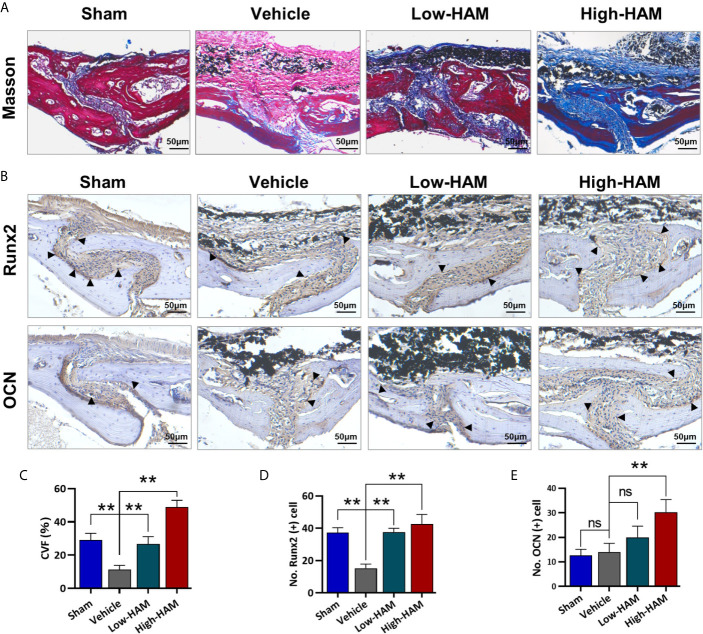
Harmine promoted osteogenic formation in the murine PPO model. **(A)** Histological staining of Masson. **(B)** Immunohistochemical staining of Runx2 and OCN. **(C)** Collagen volume fraction. **(D)** Number of Runx2 positive cells. **(E)** Number of OCN positive cells. Scale bar indicates 50 μm. n = 3. All data were expressed as the mean ± SD, ns, no significance; **p < 0.01, compared with the vehicle group.

In order to further study whether harmine treatment could enhance osteogenesis ability in Ti-particle-induced osteolysis, masson and immunohistochemical staining was performed to assess osteoblastic bone formation *in vivo*. Ti particles decreased the bone collagen fibers and collagen volume fraction (CVF) (**Figures 4A, C**). However, treatment of harmine highly improved bone collagen fiber formation and CVF in the presence of Ti particles. Based on the immunohistochemical staining, the level of critical osteogenic differentiation factor of Runx2 was obviously decreased after stimulation of Ti particles compared with the sham group (**Figures 4B, D**). In detail, 55.4% decline (p <0.01) of Runx2-positive cells number was found by Ti particles (**Figure 4D**). However, higher expression level of Runx2-positive cells was observed in the harmine-treatment groups, which is consistent with the results of immunohistochemical staining of OCN (**Figures 4B, D, E**).

### Harmine Promoted the Polarization of Macrophages From M1 to M2 *In Vitro*

**Figure 5:**
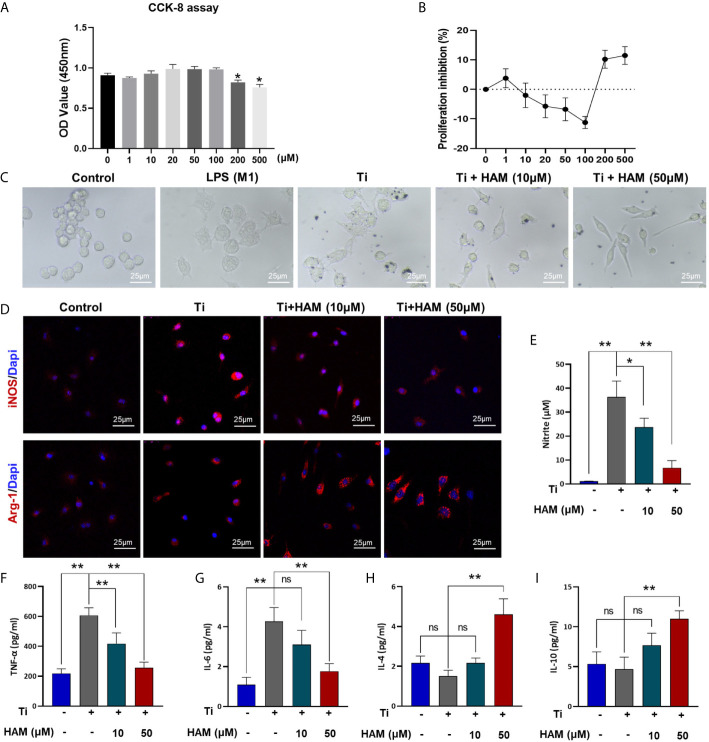
Harmine promoted the polarization of macrophages from M1 to M2 *in vitro*. **(A)** Cell proliferation was evaluated by CCK-8 assay. **(B)** Proliferation inhibition of harmine on RAW macrophages. **(C)** Morphology of RAW macrophages by a light microscope. Scale bar indicates 25 μm. **(D)** Representative immunofluorescent staining images: red (M1 marker: iNOS and M2 marker: Arg-1), and blue (Dapi, directing against nuclei). **(E)** NO content determined by Griess reagent. **(F**–**I)** Protein level expressions of inflammatory cytokines in Ti particles-treated macrophages (measured by ELISA). Scale bar indicates 25 μm. n = 3. All data were expressed as the mean ± SD, ns, no significance; *p < 0.05, **p < 0.01, compared with the Ti group.

CCK-8 assay was conducted and cell growth inhibition rate was obtained to assess toxicity of harmine for RAW264.7 cells. The data showed cell viability was unaffected by treatment with concentrations below 100 μM of harmine (**Figures 5A, B**). Cell morphological analysis was performed as shown in **Figure 5C**. The classical M1 phenotype macrophages are usually flat with multiple synaptic structures, while M2 phenotypes present spindle. In the control group without Ti particles, the majority of RAW264.7 cells appeared small and round. Lipopolysaccharide (LPS) or TNF-α is usually used as an activator of M1 macrophages (32). And the results showed LPS induced multiple M1 phenotype macrophages. And after stimulation of Ti particles, the cells were flat with many synaptic structures, which is like that in LPS group. However, harmine treatment significantly decreased the number of polygonal cells, and cone-shaped cells highly improved by the higher concentration of harmine (**Figure 5C**).

Immunofluorescence assay was carried out to evaluate the effect of harmine on the activation of macrophages *in vitro*. The results showed Ti particles enhanced the ratio of M1 macrophages (iNOS^+^, red) compared with control group (RAW in **Figure 5D** and BMDM in **Figure S2**). On the contrary, harmine treatment featured a higher expression of the M2 macrophages (Arg-1^+^, red) compared with Ti group (**Figure 5D**). And the inflammatory NO production in the supernatants was much higher in the Ti group than that in the control group. However, harmine was found to reduce Ti-induced NO production in a dose-dependent manner (**Figure 5E**). Besides, western blot analysis of RAW264.7 cells revealed that Ti particles stimulation enhanced expression of iNOS (**Figure S3**). Interestingly, harmine treatment significantly reduced iNOS expression and enhanced CD206 expression compared with that in the Ti group (**Figure S3**). These data demonstrated that harmine treatment could shift the macrophage from the proinflammatory M1 phenotype to the anti-inflammatory M2 phenotype in line with the histological study as mentioned above.

### Cytokine Production and Gene Expression *In Vitro*

**Figure 6:**
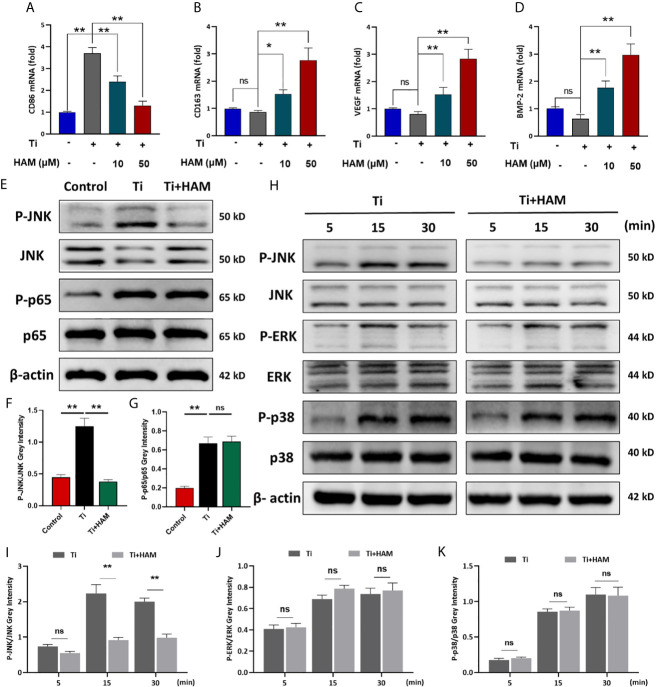
Harmine suppressed Ti particle-induced activation of JNK signaling *in vitro*. RT-PCR results for **(A)** M1 marker CD86, **(B)** M2 marker CD163, **(C)** VEGF and **(D)** BMP-2. **(E)** JNK, P-JNK, p65 and P-p65 protein levels, detected by western blot analysis. The relative levels of **(F)** P-JNK/JNK and **(G)** P-p65/p65. **(H)** JNK, P-JNK, ERK, P-ERK, p38 and P-p38 protein levels at various times, detected by western blot analysis. The relative levels of **(I)** P-JNK/JNK, **(J)** P-ERK/ERK and **(K)** P-p38/p38. n = 3. All data were expressed as the mean ± SD, ns, no significancel *p < 0.05, **p < 0.01.

As shown in **Figures 5F, G**, compared with the control group, Ti particle stimulation enhanced the inflammatory cytokine exprssion of TNF-α and IL-6. However, harmine treatment highly reduced TNF-α and IL-6 expression. On the other hand, in the high concentration harmine-treatment group, cells expressed the supreme level of IL-4 and IL-10 (**Figures 5H, I**), which are mostly secreted from M2 macrophages. RT-PCR revealed that after treatment of harmine, CD86 (M1 marker) mRNA level was downregulated compared with the Ti group (**Figure 6A**). By contrast, CD163 (M2 marker) expression was upregulated in the harmine-treatment groups (**Figure 6B**). Besides, harmine treatment enhanced BMP-2 and VEGF (bone-related gene) mRNA levels (**Figures 6C, D**), indicating a potential osteogenic ability with treatment of harmine.

### Harmine Suppressed Ti Particle-Induced Activation of JNK *In Vitro*

JNK signaling pathway has been proved to be a critical modulator of macrophage polarization and inflammatory cytokine generation (33). And macrophage polarization is also managed by nuclear factor-κB (NF-κB) (p65, IκBa) (34, 35). In this study, western blot analysis of RAW264.7 cells revealed that Ti particles stimulation obviously enhanced phosphorylation of JNK and p65 (**Figures 6E–G**). Interestingly, Ti-particle-induced activation of JNK was suppressed by harmine (**Figures 6E, F**). However, activation of p65 was unaffected with harmine treatment (**Figures 6E, G**). To further identify the inhibitory effect of harmine on the JNK signaling pathway, after pre-treatment with harmine for 4 h, cells were stimulated with Ti particles for various times (5, 15, or 30 min). JNK is one of three major mitogen-activated protein kinases (MAPKs) signaling pathways (p38, ERK, and JNK). The data revealed that harmine had no inhibitory effect on p38, ERK and p65 (**Figures 6H, J, K** and **Figure S4**). Nevertheless, JNK activation in the presence of Ti particles was significantly inhibited in the harmine treated group (**Figures 6H, I**). These data indicate that harmine reduced particle-induced inflammation and that JNK might play a central role in this process.

### The Assessment of Osteogenic Differentiation Ability in Conditioned Medium

**Figure 7:**
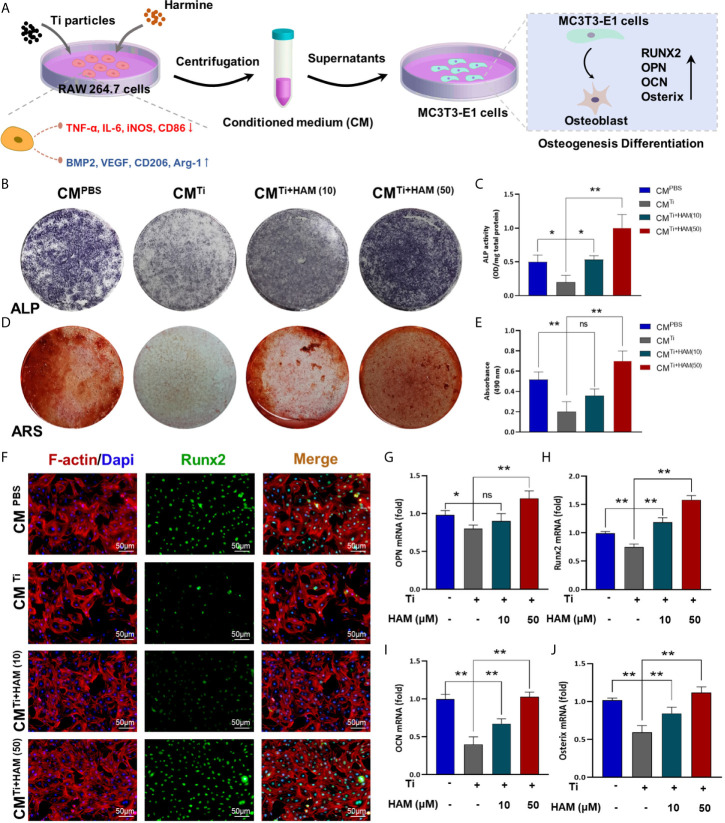
The assessment of osteogenic differentiation ability in conditioned medium. **(A)** Pattern diagram. We used the supernatants of RAW264.7 cell culture as conditioned medium to assess immunomodulatory effect of harmine on osteogenic differentiation in MC3T3-E1 cells. **(B)** ALP staining assessed osteogenic differentiation of MC3T3-E1 cells in different groups. **(C)** ALP activity. **(D)** ARS staining for measuring mineralization of MC3T3-E1 cells from each group. **(E)** Absorbance at 490 nm. **(F)** Images of the MC3T3-E1 cells after coimmunostaining: red (actin rings), green (Runx2) and blue (nuclei). Scale bar indicates 50 μm. RT-PCR results for **(G)** OPN, **(H)** Runx2, **(I)** OCN and **(J)** Osterix in conditioned medium from different groups. n = 3. All data were expressed as the mean ± SD, ns, no significance; *p < 0.05, **p < 0.01, compared with the Ti group.

We used the supernatants of RAW264.7 cell culture as conditioned medium to assess immunomodulatory effect of harmine on osteogenic differentiation in MC3T3-E1 cells (**Figure 7A**). After the cells were cultured in CM, ALP staining and ARS staining were performed as mentioned above. The ALP activity in the CM^Ti^ group was 58.5% (p <0.05) lower than the CM^Control^ group (**Figures 7B, C**). However, ALP activity was greatly increased in the CM^Ti + harmine10^ and CM^Ti + harmine50^ groups. Meanwhile, ARS staining revealed mineralization was decreased in the CM^Ti^ group (**Figures 7D, E**). Greater calcium mineralization was observed in the CM^Ti + harmine50^ group, followed by the CM^Ti + harmine10^ group. On the side, immunofluorescence staining was carried out and a similar tendency was found: the CM^Ti + harmine50^ group had the highest expression of Runx2, while the lowest Runx2 expression appeared in the CM^Ti^ group (**Figure 7F**). Meanwhile, RT-PCR was performed to assess the impact of CM on osteogenic transcription factors. Similarly, the mRNA levels of OPN, Runx2, OCN and Osterix were decreased in the CM^Ti^ group (**Figures 7G–J**). However, the CM^Ti + harmine50^ group had a higher mRNA levels of these osteogenic transcription factors.

## Discussion

TJA is a well-established effective treatments for terminal joint sickness in past decades. Increasing evidence indicates that wear particles, generated from joint prostheses, can induce a chronic inflammation response by the further recruitment of macrophages, which secrete multiple proinflammatory factors, such as chemokines and cytokines (36, 37). And it has been demonstrated that macrophage polarization exerts great influence in the pathogenesis of inflammatory osteolysis. Therefore, targeted suppression of inflammatory response and macrophage polarization is clearly a potential treatment for alleviating wear-particle-induced bone loss after TJA.

Recently, harmine, a β-carboline alkaloid, has been reported to reduce bone loss of osteoporotic mice and affect bone metabolism. Furthermore, it has been reported that harmine inhibits osteoclast differentiation and promotes osteoblast differentiation through bone morphogenetic protein signaling pathway (26–28). Moreover, the immunomodulatory effects of harmine have also drawn much attention. Nevertheless, to this day, the effect of harmine on PPO and macrophage polarization has not been demonstrated. So, this experiment was conducted to investigate whether harmine could alleviate debris-irritated bone loss in a murine osteolysis model. As previously reported, Ti particles can induce severe bone destruction in the murine calvaria (38). To our expectation, harmine greatly attenuated the severity of bone destruction in debris-irritated mice. Micro-CT revealed that BMD and BV/TV were enhanced and porosity was reduced by application of harmine. And histomorphological staining ulteriorly showed that harmine treatment attenuated Ti particle-irritated osteolysis and reduced eroded bone surface. Collectively, these evidences indicated that treatment with harmine might produce an active effect to treat PPO. However, it has been reported that intraperitoneal injection of harmine could bring some side effects (25, 39), which slow the process of harmine toward wider clinical applications. During the experiment, harmine emulsion was used and all the mice oral gavaged with harmine emulsion survived and did not have any side effects. And oral administration is more convenient and acceptable in clinic. Therefore, harmine emulsion may be a hopeful and more secure therapeutic agent for osteolytic disease, which requires verification by future clinical trials.

Macrophages have multiple functions and play an important role in response to microenvironmental signals. For the past few years, the concept of M1 macrophages and M2 macrophages has been built. Specifically, M1 phenotype is proinflammatory, while M2 phenotype is anti-inflammatory (13, 14). Wear particles around the prosthesis have been reported to enhance M1 macrophages expression and lower expression of M2 macrophages. Regulation of the macrophage polarization could bring benefit for attenuating wear debris-irritated inflammation. And whether harmine attenuated Ti particle-irritated osteolysis by regulating macrophage polarization remains unknown. Immunohistochemical staining of calvarial tissue showed the inflammatory cytokines of IL-1β, TNF-α, and IL-6 were greatly elevated by stimulation of Ti particles. On the contrary, the level of these inflammatory cytokines was lowered with harmine treatment. This indicates that harmine could inhibit particle-induced inflammatory response. As previously described, stimulation of Ti particles enhanced the expression of F4/80^+^/iNOS^+^-positive M1 phenotype and lowered F4/80^+^/Arg-1^+^-positive M2 phenotype expression. Nevertheless, penetration of M1 phenotype macrophages is reversed by harmine and the proportion of anti-inflammatory M2 phenotype macrophages increases. This indicated harmine treatment may shift the polarization of macrophages from M1 to M2, thereby decreased inflammation in PPO. Meanwhile, the effect of harmine on macrophage polarization was investigated *in vitro*. Cell morphological analysis of macrophages revealed that Ti particles induce classical M1 phenotypes with numerous synaptic structures, while harmine significantly decreased the number of M1 macrophages and increased the number of M2 macrophages, especially in the high-harmine concentration group. In addition, the results of immunofluorescence assay and western blot analysis directly demonstrated that harmine increased Arg-1 level and decreased iNOS level. In addition, variations in proinflammatory and anti-inflammatory cytokines revaled the diversity between M1 and M2 phenotypes. As expected, RT-PCR results were consistent with immunofluorescence results, harmine treatment decreased the mRNA level of CD86 and enhanced CD163 expression. In general, these data demonstrated that harmine could inhibit particle-induced inflammatory response and shift macrophages from proinflammatory M1 phenotypes to anti-inflammatory M2 phenotypes.

As previously reported, unnatural bone remodeling in the inflammatory microenvironment is similarly crucial in particle-irritated bone destruction (40, 41). Increasing evidence have indicated that wear debris can suppress osteogenic differentiation in osteolysis (42, 43). Bone formation plays a critical role in treating osteolytic disease. Low expression of M2 macrophages in the particle-induced inflammatory microenvironment had a negative effect on osseointegration and angiogenesis (16, 17). In this experiment, we proved that harmine could reverse the decline in bone collagen fibers under the particle-stimulated inflammatory condition, and enhance the levels of critical osteogenic differentiation factors of Runx2 and OCN *in vivo*. Besides, BMP-2 and VEGF mRNA levels were also increased in the harmine-treatment groups *in vitro*, indicating a potential osteogenic ability with treatment of harmine. Moreover, ALP staining, ARS staining and immunofluorescence staining of MC3T3-E1 cells cultured in CM further proved that harmine could promote subsequent osteogenic differentiation capacity in an inflammatory microenvironment. Additionally, the immunomodulatory correlation between macrophage polarization and osteogenesis has been reported to be a critical mechanism involved in bone regeneration (44–46). It has been suggested that harmine could affect bone metabolism as described above. Indeed, the direct relationship between harmine and osteoblastogenesis as well as osteoclastogenesis has not been well understood in PPO, which needs further investigation in future studies.

We subsequently explore the underlying mechanism of harmine-induced suppression on local inflammation as well as regulation of macrophage polarization. It has been proved that M1 phenotype macrophages stimulated by wear particles could produce a continuing inflammatory reaction *via* the JNK signaling pathway (33). The JNK can be phosphorylated and induce downstream gene transcription. As previously described, JNK signaling pathway could be activated in bone marrow-derived macrophages by stimulation of wear debris. JNK is crucial to the activation of the c-jun transcription factor and was reported to accelerate osteoclastogenesis (36, 47). And macrophage polarization is also regulated by NF-κB signaling pathway (34, 35). Western blotting suggested that JNK might play a central role in regulating macrophage polarization and inflammatory reactions. JNK signaling pathway is usually excitated by two upstream mitogen-activated protein kinase kinases (MAP2Ks) (MKK4 and MKK7), which straightly phosphorylate JNK on threonine (Thr183) and tyrosine (Tyr185) residues (48). Besides, MKK4 and MKK7 are activated by upstream pathways *via* multiple mitogen-activated protein kinase kinase kinases (MAP3Ks) (49). Nevertheless, the accurate molecular mechanisms involved in the regulation of inflammation and macrophage polarization by harmine require further investigation in the future.

There were some limitations in our study. First, the murine calvarial model in this experiment produced acute inflammation rather than the chronic inflammation generally existed in aseptic loosening. The osteolysis process was investigated for a short period of 14 days. Then a larger animal model more be more suitable for assessing the long-term efficacy and safety of harmine treatment. Secondly, Ti particles were used in the study rather than ultrahigh molecular weight polyethylene debris, which are the primary materials to induce osteolysis in clinical practice (50). We chose Ti particles here because of their stability and easy adhesion. Ti particles and polyethylene debris have been reported to trigger osteolytic effects comparably (51, 52).

## Conclusion

**Figure 8:**
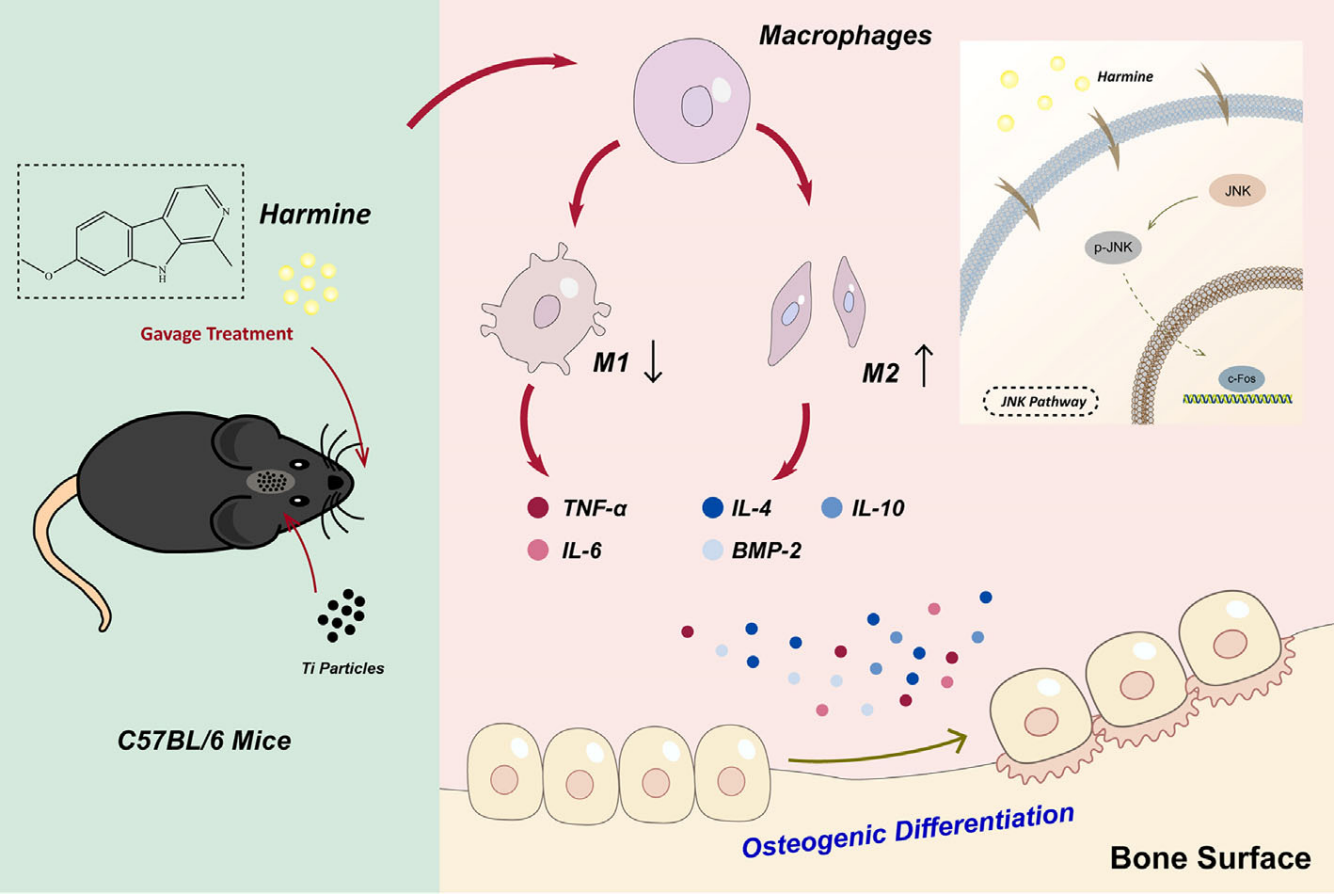
Harmine alleviated the particle-induced inflammation and facilitated the polarization of macrophages from M1 phenotypes to M2 phenotypes by blocking the JNK signaling pathway as well as osteogenic formation *in vitro* and *in vivo*.

In general, this study indicated that in wear debris-irritated inflammatory osteolysis, harmine notably alleviated the particle-induced inflammatory response and shifted macrophage polarization from M1 phenotype to M2 phenotype as well as osteogenic formation *in vitro* and *in vivo*. And the immunomodulatory ability of harmine might be attributed to the suppression of JNK signaling pathway (**Figure 8**). In addition, the findings demonstrated for the first time that harmine exerts a protective influence on a murine PPO model. All the results strongly show that harmine can be a promising therapeutic agent to treat PPO.

## Data Availability Statement

The original contributions presented in the study are included in the article/**Supplementary Material**. Further inquiries can be directed to the corresponding authors.

## Ethics Statement

The animal study was reviewed and approved by Ethics committee of Changzhou No. 2 Hospital.

## Author Contributions

LW conducted all the experiments with assistance from the other authors. GG, WW and QW assisted in the surgical procedure of establishing murine calvarial osteolysis model and biochemical analysis. NX and GG performed the histological staining and analysis. DZ and SJ performed the cell culturing, differentiation, qRT-PCR and western blot assay in vitro. GZ conducted the Micro-CT scanning and reconstruction analysis. YX oversaw the experiments. YW and RZ provided academic guidance suggestions. DG wrote the manuscript with contributions from LW and QW. All authors contributed to the article and approved the submitted version.

## Funding

This research was supported by the National Natural Science Foundation of China (82002321, 82072425, 81873991), the Social Development Project of Jiangsu Province (BE2015632). the Jiangsu Provincial Medical Youth Talent (QNRC2016751), the Natural Science Foundation of Jiangsu province (BK20180001), and Special Project of Diagnosis and Treatment for Clinical Diseases of Suzhou (LCZX202003).

## Conflict of Interest

The authors declare that the research was conducted in the absence of any commercial or financial relationships that could be construed as a potential conflict of interest.
