# Supplementary material for: Harmine Alleviates Titanium Particle-Induced Inflammatory Bone Destruction by Immunomodulatory Effect on the Macrophage Polarization and Subsequent Osteogenic Differentiation
Source: Front Immunol. 2021 May 17;12:657687. doi: 10.3389/fimmu.2021.657687 (PMC8165263; doi:10.3389/fimmu.2021.657687)
Supplement: Supplementary file 1 [file DataSheet_1.docx]

**Supplementary Information for**

Harmine alleviates titanium particle-induced inflammatory bone destruction by immunomodulatory effect on the macrophage polarization and subsequent osteogenic differentiation

**The name(s) of the author(s):**

Liangliang Wang^1,†^; Qing Wang^2,†^; Wei Wang^2,†^; Gaoran Ge^2^; Nanwei Xu^1^; Dong Zheng^1^; Shijie Jiang^1^; Gongyin Zhao^1^; Yaozeng Xu^2^; Yuji Wang^1,3,4,*^; Ruixia Zhu^1,*^; Dechun Geng^2,*^

**Name and address of the institution:**

1: Department of Orthopaedics, the Affiliated Changzhou No.2 People’s Hospital of Nanjing Medical University, Changzhou, P. R. China;

2: Department of Orthopaedics, the First Affiliated Hospital of Soochow University, Suzhou, P. R. China;

3: Departments of Orthopedic Surgery and Biochemistry and Molecular Biology, Mayo Clinic, Rochester, Minnesota.

4: Department of Orthopedics, the Third Affiliated Hospital of Gansu University of Chinese Medicine, Baiyin, P. R. China.

**Corresponding authors:**

**Yuji Wang**

Department of Orthopaedics, the Affiliated Changzhou No.2 People’s Hospital of Nanjing Medical University, 29 Xinglong Alley, Changzhou, 213003, China.

E-mail: [yujiwang@sohu.com](mailto:yujiwang@sohu.com);

**Ruixia Zhu**

Department of Orthopaedics, the Affiliated Changzhou No.2 People’s Hospital of Nanjing Medical University, 29 Xinglong Alley, Changzhou, 213003, China.

E-mail: 392849984@qq.com;

**Dechun Geng**

Department of Orthopedics, The First Affiliated Hospital of Soochow University, 188, shi zi Road, Suzhou, 215006, China.

E-mail: [szgengdc@163.com](mailto:szgengdc@163.com).

^†^ **Contribute equally to this work**

**
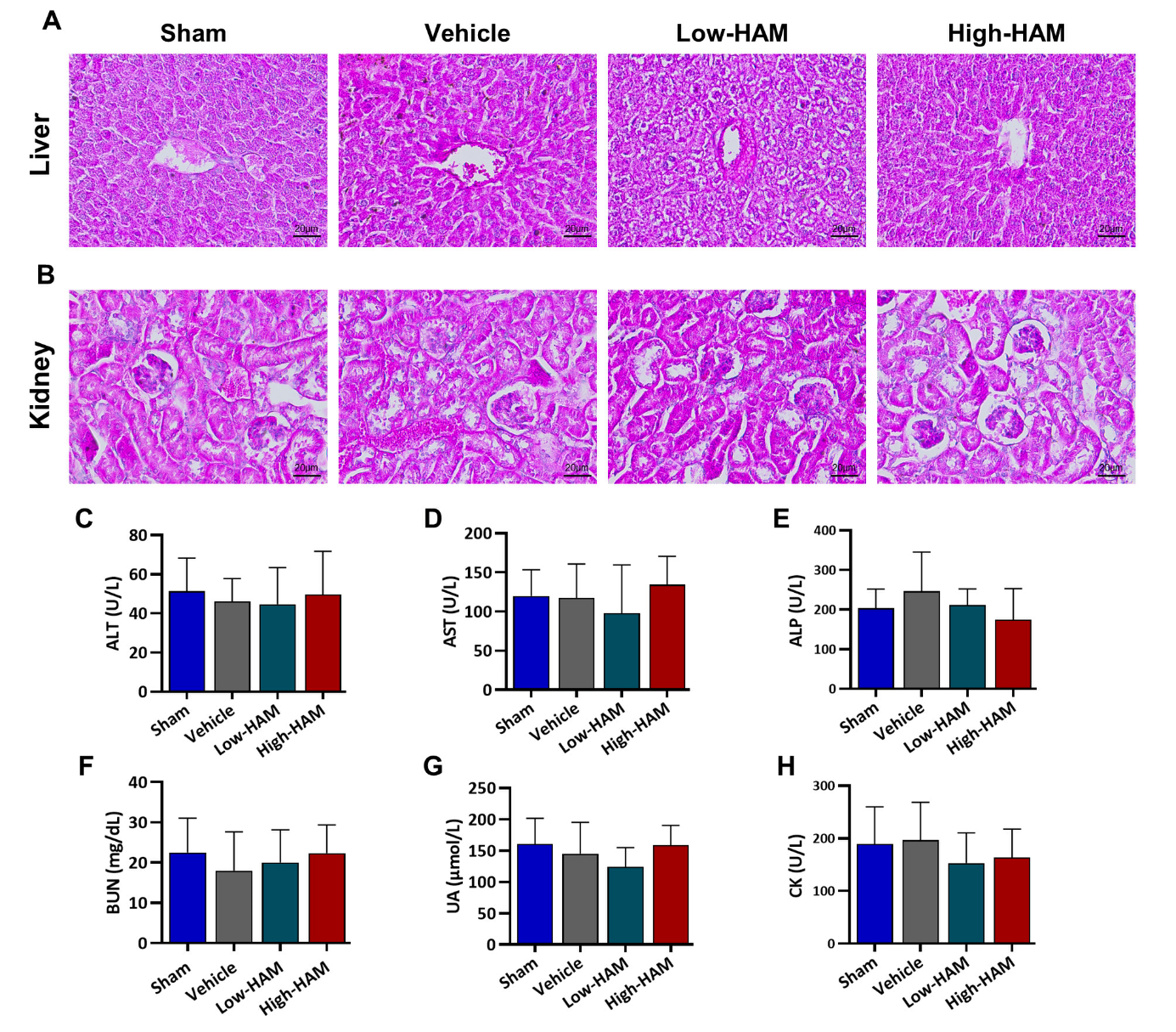
**

**Fig. S1.** Harmine has no toxicity for the mice in vivo. H&E staining of (A) liver and (B) kidney. Biochemical index evaluation of blood samples: (C) ALT, (D) AST, (E) ALP, (F) BUN, (G) UA and (H) CK. All indexes are in normal range. n = 3. All data were expressed as the mean ± SD.

**
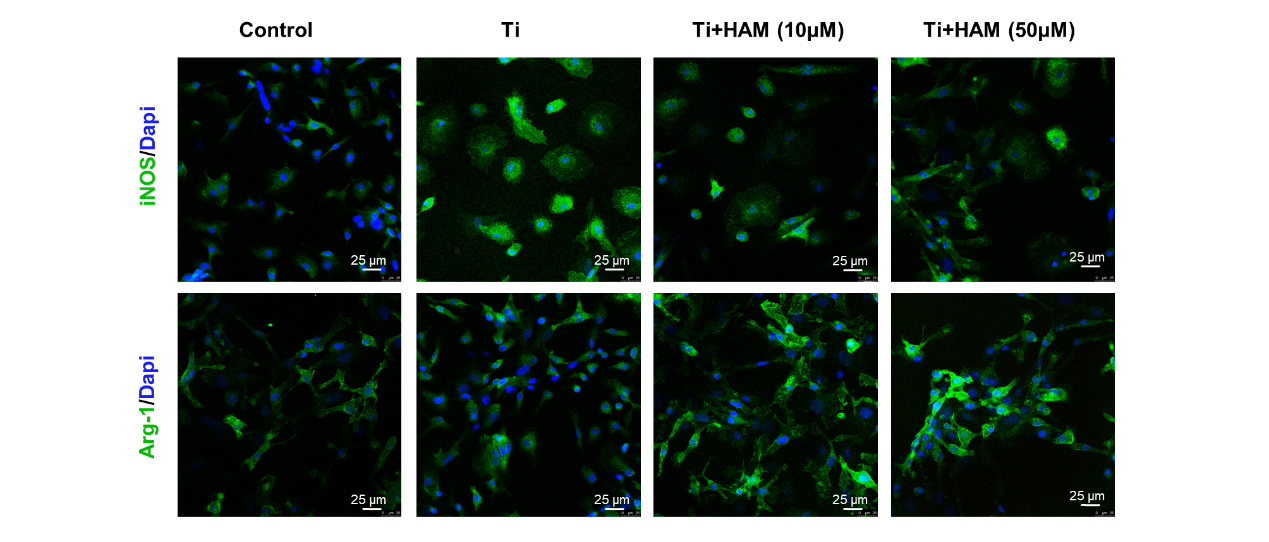
**

**Fig. S2.** Harmine promoted the polarization of macrophages from M1 to M2 in BMDM. Representative immunofluorescent staining images: green (M1 marker: iNOS and M2 marker: Arg-1), and blue (Dapi, directing against nuclei). n=3.

**
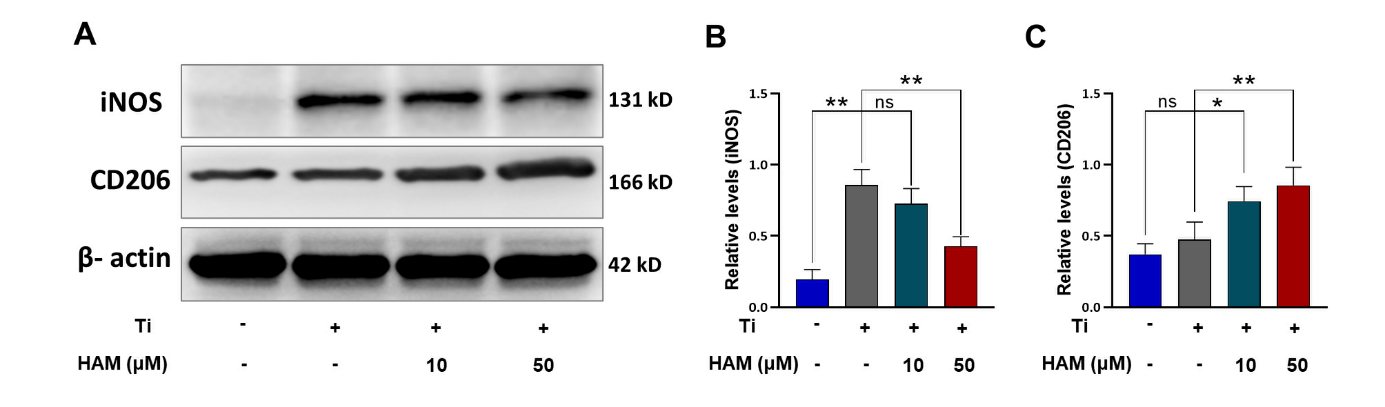
**

**Fig. S3.** Harmine reduced iNOS expression and enhanced CD206 expression in vitro. (A) iNOS and CD206 protein levels, detected by western blot analysis. (B) The relative levels of iNOS and CD206. n = 3. All data were expressed as the mean ± SD, ns. no significance, *p < 0.05, **p < 0.01.


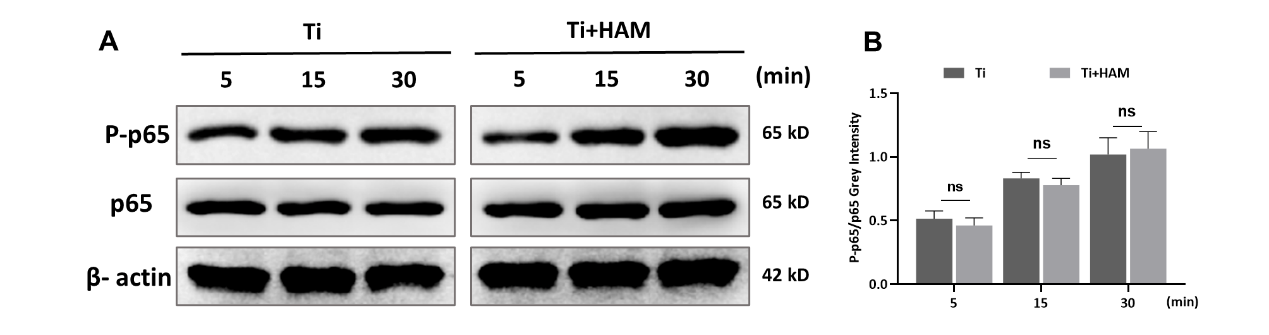


**Fig. S4.** Harmine did not suppress Ti particle-induced activation of NF-κB signaling in vitro. (A)P-p65 and p65 protein levels at various times, detected by western blot analysis. (B) The relative levels of P-p65/p65. n=3. All data were expressed as the mean ± SD, ns. no significance.
